# Comparing Direct Observation of Torsion with Array-Derived Rotation in Civil Engineering Structures

**DOI:** 10.3390/s21010142

**Published:** 2020-12-28

**Authors:** Philippe Guéguen, Frédéric Guattari, Coralie Aubert, Theo Laudat

**Affiliations:** 1 ISTerre, Université Grenoble Alpes, USMB, CNRS, IRD, Université Gustave Eiffel, 38058 Grenoble, France; coralie.aubert@univ-grenoble-alpes.fr; 2 iXblue, 78100 Saint-Germain-en-Laye, France; frederic.guattari@ixblue.com (F.G.); theo.laudat@ixblue.com (T.L.)

**Keywords:** rotation, array-derived, civil engineering, buildings, structural health monitoring (SHM), City-Hall Grenoble

## Abstract

In this article, we analyze the rotation rates in a building derived from a network of translation sensors and recorded by a rotation sensor. The building is Grenoble city hall, a reinforced concrete structure with permanent accelerometric translation sensors at the top and bottom of the building. A temporary experiment was conducted, consisting in installing a BlueSeis-3A rotation sensor for more than 24 h at the top of the structure. The ambient vibrations were analyzed. The amplitudes of translation accelerations and rotation rates at the top and bottom of the building, along with their variations over time, were analyzed. The acceleration/rotation ratios were then compared with the impulse wave velocities derived from seismic interferometry by deconvolution between the top and bottom. Perspectives with regard to building imaging, time monitoring of structural integrity and understanding the contribution of rotations to the structure’s response are discussed, offering new suggestions for research projects.

## 1. Introduction

This study examines the opportunities offered by the use of rotation sensors in civil engineering structures for non-parametric modal analysis of a building’s elastic rotation response. The structural response in rotation is likely to be an essential element to be considered since rotation can modify the response of a large majority of structures during earthquakes. In particular, rotation about vertical axis (torsion) can cause an increase in the forces and stresses exerted on the structural elements, thus becoming a critical element in structural design (e.g., [1,2]). This rotation can be caused by a static component related to the eccentricity between the centres of mass and rigidity, particularly in asymmetrical structures [1,3,4]. There is also a so-called accidental component in symmetrical and asymmetrical buildings, which can, among other things, be due to a difference between theoretical and real design resulting in increased eccentricity, dynamic effects depending on the levels of loading or the effects of rotational ground motion at the bottom of the building [5,6,7,8]. For this last component, the dynamic properties of structures (i.e., resonance frequency in translation and ratio of translation to rotation frequencies Ω) modify the impact of the rotational motion on the structural response (e.g., [9,10,11,12]).

Several experiments show the importance of the torsion response of buildings in damage distribution [13,14,15,16] even in buildings of apparently symmetrical design. In fact, a clear torsion mode is more that common observed in most modal analysis studies based on ambient vibrations, regardless of structure asymmetry (examples among many others, [17,18]). However, Anagnostopoulos et al. [2] report that the treatment of vertical axis rotation by modern seismic codes varies considerably due to simplifications considered for this complex issue. Most studies on torsion are, in fact, mainly based on numerical models.

Pure rotation sensors have only recently become available and without reliable direct measurements, indirect methods for engineering purposes have been designed using translation sensors. The use of sensor arrays has probably become the most popular solution, consisting in calculating spatial derivatives of translation components motion based on a finite difference scheme (e.g., [19,20,21]). Many experimental studies in buildings applied this method to estimate the rotation rate of building responses (e.g., [22,23,24,25]). Lin et al. [26] recently proposed a comparison between array-derived rotation and point rotation in 101 Taipei tower, which is probably the only comparable study, since relatively few rotation sensors have been deployed in structures. However, this brings the question of knowing to what extent the hypotheses applicable to seismology (infinitesimal strain, semi-infinite space, etc.) can be transferred to structures, also bearing in mind that the inter-station distances of arrays are imposed by the horizontal dimensions of the structures and that the number of sensors deployed is relatively limited.

Moreover, experiences in the lab with microelectromechanical systems (MEMS) inclinometers for inter-story drift assessment [27], numerical analyses of beam-like bridge loaded with moving point load [28] or on-site analysis of progressive damage bridge case studies with different rotation measurements confirmed the sensitivity of rotation as a parameter for damage identification (e.g., among others [29,30,31]) that open new perspectives for seismic structural health monitoring of civil engineering buildings. 

Lee and Trifunac [32] thus stated that despite recurrent engineering studies demonstrating the importance of rotations in the building response, the development and deployment of rotation sensors have progressed relatively slowly in the field of earthquake, particularly in structures. However, a new generation of rotation sensors has emerged in the past decade, a development notably motivated by seismology [33] and the significant contribution of rotational motion to the description of the seismic wave field and imaging of the internal structure of the earth (e.g., [34,35]). The purpose of this paper is therefore to present a real case study on the use of rotation sensors in a structure, particularly to identify the torsion mode using a single sensor for earthquake engineering and Seismic Structural Health Monitoring fields.

This paper is organized as follows: In Section 2, Grenoble city hall building (GCH) is described along with the experimental translation data acquired by the permanent array in GCH, and the data recorded during a temporary experiment performed with a rotation sensor (BlueSeis-3A). The processing of translation and rotation data is described in Section 3. The results are presented in Section 4. The time variations of the translation and torsion motions are discussed first, then the array-derived rotation is compared with the rotation sensor measurement, followed by the relationships between acceleration and rotation. These relationships are then compared in an attempt to estimate the phase velocity of the beam-like building. Finally, the conclusions of this study are presented.

## 2. Data

**Figure 1:**
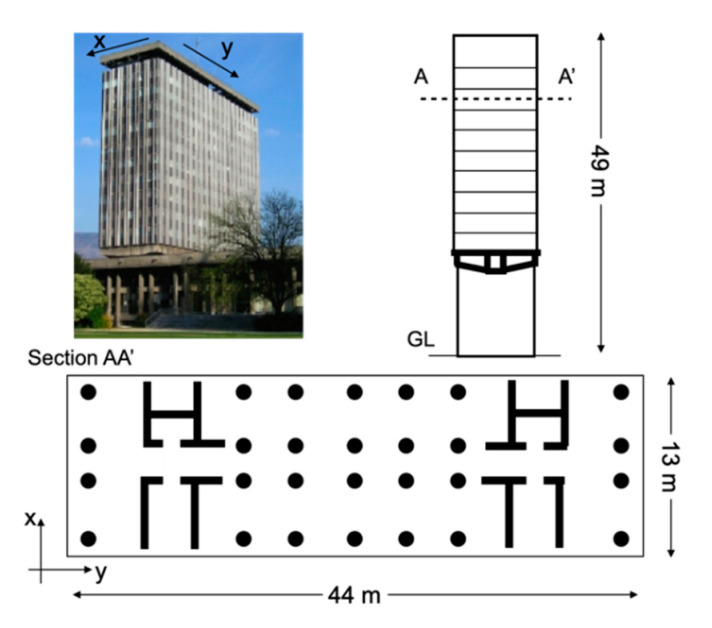
Representation of the structure of Grenoble city hall (GCH).

The concerned building (GCH) is Grenoble city hall. GCH is a reinforced concrete structure that was completed in 1967 (Figure 1), a full description of which is provided in [18]. The structural elements are made up of continuous reinforced concrete shear walls, with the stairwells and lift shafts at the two ends of the building. A prestressed slab with a span of 23 m on the second floor supports the floors above. The structural resistance system combines shear walls, reinforced concrete columns and reinforced concrete longitudinal beams to support the floors. The outside of the building is covered with a glazed frontage attached to a light steel frame. Michel et al. [18] performed the modal analysis of the structure using ambient vibration measurements. They identified the three primary vibration modes: the first longitudinal mode (direction y) at 1.16 Hz, the first transverse mode (direction x) at 1.22 Hz, and the first torsion mode at 1.44 Hz. Considering the design and lateral resonance frequencies, we can assume that the building is symmetrical, i.e., low static eccentricity between the centres of mass and rigidity. Modal analysis enables consideration of the uncoupled translation and torsion modes. The ratio of uncoupled frequencies between torsion and lateral vibration (Ω) is 1.18 (direction x) and 1.24 (direction y), considering the building as a torsionally-stiff structure [9,12], assumed by these authors to imply a minor contribution of accidental eccentricity due to ground motion rotation.

The building is built in a very deep sedimentary basin [36]. The basin is filled with soft lacustrine deposits, approximately 900 m deep in the centre of the valley, with a shear wave velocity (Vs) gradient of approximately 300–900 m/s [37]. In the immediate vicinity of GCH, there is a superficial layer of very soft (Vs approximately 200 m/s) clay and peat deposits, covering a stiff layer of gravel and sand at a depth of 12 m, resulting in a site effect of approximately 2 Hz. Michel et al. [18] and Guéguen et al. [38] confirmed the influence of the soil-structure interaction on the structural response, shifting the resonance frequency of the soil-structure system towards the low frequencies.

**Figure 2:**
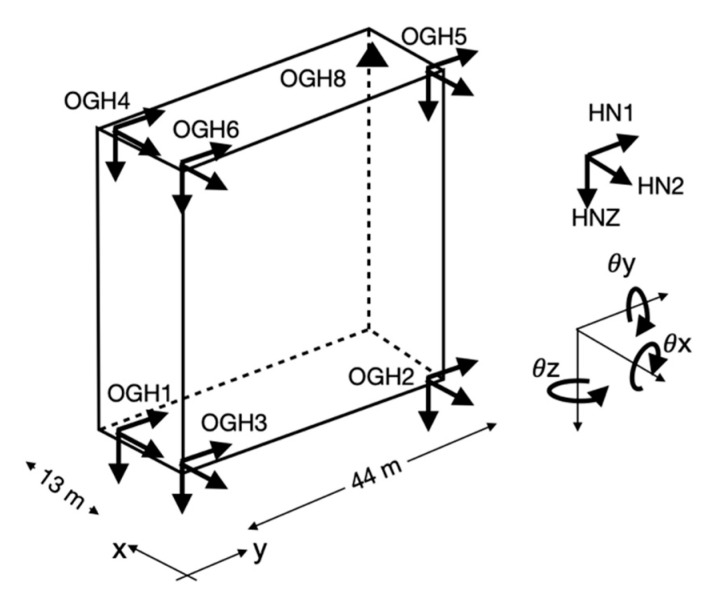
Description and representation of the instrumentation deployed in GCH and identification of the translation and rotation components.

**Figure 3:**
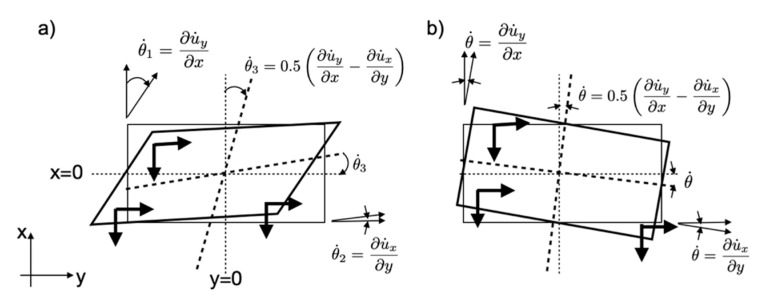
Schematic view of the rotations calculated by the array (**a**) in the case of shear deformation and (**b**) in rigid body conditions.

The building is monitored since 2004 by the French Accelerometric Network (RAP-RESIF) [39]. Three accelerometric stations on the ground floor, called OGH1, OGH2 and OGH3, and three on the 13th floor (roof), called OGH4, OGH5 and OGH6 (Figure 2), continuously record the building’s vibrations, and send the data in real time to the French Seismilogical network (RESIF) datacentre hosted by Grenoble university (http://seismology.resif.fr). Each station has one 3C Episensor high sensitivity accelerometer (full scale = 1 g), oriented in the longitudinal (y, HN1 code in compliance with the International Federation of Digital Seismograph Networks (FDSN) norm) and transverse (x, HN2 code) directions, connected to a 24-bit digital acquisition system. The full-scale acquisition dynamic combined with the high sensitivity of the sensors enables recording of ambient vibrations in the urban environment of the building. The sampling frequency is 100 Hz, synchronization is coordinated by GPS and the stations are perfectly synchronized with one another. In 2019, a weather station OGH8 with GPS time synchronization was installed at the top of the structure to complete the system (Figure 2). Among the parameters collected, those used in this study are wind speed and air temperature. Using the permanent array, Michel [40] also performed a least-squares inversion of the translation components at the top to find the centre of the rigid-body rotation. He observed a shift of the centre of rotation (−0.63 ± 0.15 m; 1.50 ± 0.07 m) in directions x and y (indicated in Figure 3), i.e., a slight shift compared with the geometrical centre of the structure, without accurately evaluating the centres of mass and rigidity.

From 1 October 2019 to 2 October 2019, a blueSeis-3A rotational motion broadband sensor from iXblue (www.blueseis.com) was temporarily installed near sensor OGH6 to provide the rotation rate of the vibration $\dot{\theta }$ (Figure 2). The sensor was simply placed on the slab, protected against weather conditions, based on the assumption that its mass would ensure good coupling with the structure under ambient vibrations. The high theoretical sensitivity of the blueSeis-3A (2.5 × 10^−8^ rad/s–0.5 rad/s in the frequency range 0.01–50 Hz) and its flat transfer function between 0 (i.e., DC) and a few kHz mean that this sensor can be used as a single point of measurement for rotation. However, its position near OGH6 in one corner of the building was determined to guarantee sufficiently accurate rotation measurement above the noise, without a priori information on the amplitudes expected. In this study, only rotation around the vertical axis ${\dot{\theta }}_{z}$ (i.e., the building torsion response, ${\dot{\theta }}_{HJZ}$ according to the FDSN norm) is considered.

## 3. Data Processing

Assuming a structure with rigid slabs and foundations, we can neglect the in-plane deformations of the structure. Generally speaking, rotation motion can be calculated from the translation components of a pair of stations, according to a finite difference scheme (Figure 3), assuming infinitesimal shear deformation [20,23]:

(1) $${\dot{\theta }}_{z}=0.5\left(\frac{{\dot{u}}_{1y}\left(t\right)-{\dot{u}}_{2y}\left(t\right)}{\Delta x}-\frac{{\dot{u}}_{1x}\left(t\right)-{\dot{u}}_{2x}\left(t\right)}{\Delta y}\right)=0.5\left(\frac{\partial {\dot{u}}_{y}\left(t\right)}{\partial x}-\frac{\partial {\dot{u}}_{x}\left(t\right)}{\partial y}\right)$$

where ${\dot{u}}_{1}\left(t\right)$ and ${\dot{u}}_{2}\left(t\right)$ are the translation velocity (m/s) (for components x or y) recorded at the stations located at 1 and 2, separated by a distance of Δx and Δy. ${\dot{\theta }}_{z}$ is the rotation rate in rad/s.

The rotation rate, assumed to be uniform between site 1 and 2, is therefore the average rotation between points 1 and 2. The closer the stations, the closer the rotation value will be to the actual rotational gradient, but in this case, instrumental noise will have a greater impact on gradient uncertainty. Note that the translation motion induced by rotation may affect the array-derived rotation value, which will be ignored herein. Furthermore, the distance between the stations must be large enough to limit spatial aliasing and conserve a uniform gradient from one station to another. A distance less than 1/4 of the wavelength considered is generally accepted [41,42]. In our study, the resonance frequencies of the building are >1 Hz, and the shear wave velocity is around 200 m/s, which means 1/4 wavelength is about 50 m, which is more than the maximum distance between the stations (44 m in direction y, 13 m in direction x). In this study, Equation (1) rotation will be calculated with pairs OGH4-OGH5 (${\dot{\theta }}_{45}$) at the top and OGH1-OGH2 (${\dot{\theta }}_{12}$) at ground level.

When shear deformation is zero (or very slight compared with rotation), each level behaves like a rigid body (Figure 3b). The rotation rate can be calculated between any pair of stations aligned along the main axes of the structure by the relationship derived from Equation (1) (e.g., [9,22]):

(2) $${\dot{\theta }}_{z}=\frac{\partial {\dot{u}}_{y}\left(t\right)}{\partial x}=\frac{\partial {\dot{u}}_{x}\left(t\right)}{\partial y}$$

In this study, and in view of the array configuration, only the rotations derived from the translation components along y between sensors 4 and 6 (${\dot{\theta }}_{46}$) and along x between sensors 5 and 6 (${\dot{\theta }}_{56}$), are calculated (and, by association, at ground level ${\dot{\theta }}_{13}$ and ${\dot{\theta }}_{23}$).

The accelerometric translation data are taken from the permanent array during the period of the temporary experiment, i.e., approximately 24 h. The synchronised signals are divided into 10 min windows, i.e., 143 windows, from which the average and tendency are removed. The signals are band-pass Butterworth filtered between 0.5 and 5 Hz. The velocities are derived from the accelerations by simple integration.

**Figure 4:**
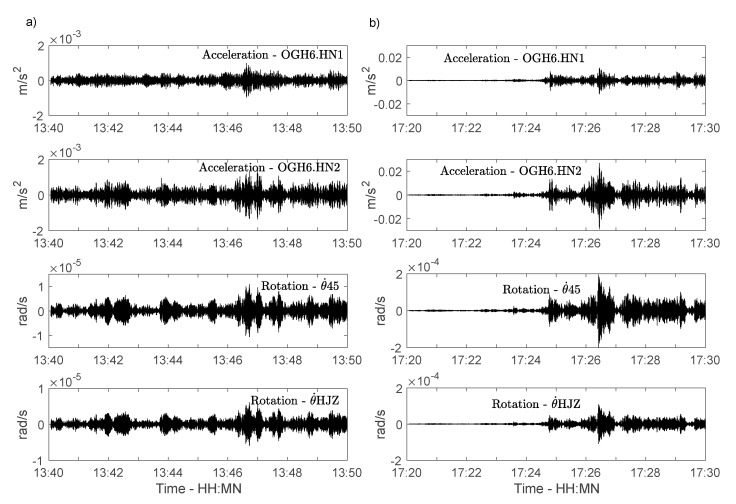
Example of 10 min recordings in translation and in rotation, (**a**) under ambient vibrations, (**b**) at the time of the local storm. Note the amplitude difference on the y axes.

Figure 4 shows an example of two 10 min windows, typical of an ambient vibration window (4a) and at the time of a sudden moderate storm occurring during the experiment (4b), which generated accelerations 10 times stronger at the top. An amplitude difference is also observed between the rotation rate according to Equation (1) (i.e., ${\dot{\theta }}_{45}$) and that provided by the rotation sensor (i.e., ${\dot{\theta }}_{HJZ}$). The magnitude-squared coherence between ${\dot{\theta }}_{45}$ and ${\dot{\theta }}_{HJZ}$ is calculated as the ratio of the products of the power spectral densities of the two signals, with the cross power spectral density of the two signals. Considering a torsion frequency of 1.44 Hz, the average of the 10 min windows gives a coherence of 0.91 between 1.42 and 1.46 Hz, considering measurements coherent in rotation. The amplitude difference will be discussed below.

**Figure 5:**
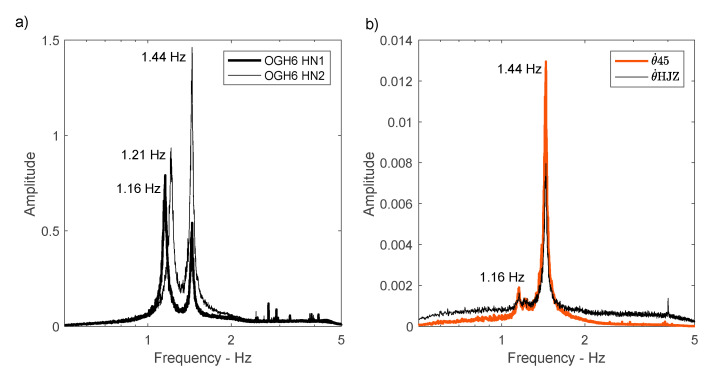
Averaged Fourier spectra of the 10 min windows. (**a**) acceleration in translation at station OGH6; (**b**) rotation rate obtained by rotation sensor HJZ and derived from the array using OGH4 and OGH5 stations (Equation (1)).

The averaged Fourier spectra recorded in translation and rotation at station OGH6 are shown in Figure 5. The translation spectra (Figure 5a) show the same resonance frequency values as [18], i.e., 1.16 and 1.21 Hz along x and y, respectively. The rotation frequency at 1.44 Hz is visible on the translation components, more marked in direction y. The rotations ${\dot{\theta }}_{45}$ and ${\dot{\theta }}_{HJZ}$ (Figure 5b) provide a very similar average spectrum, with the 1.44 Hz frequency visible and a frequency of 1.16 Hz resulting from the coupling with the translation components. ${\dot{\theta }}_{HJZ}$ measures rotation in the same place as station OGH6, at one end of the building, where the amplitude of the rotation rate is generally higher, while ${\dot{\theta }}_{45}$ estimates an average rotation between stations OGH4 and OGH5, i.e., passing through the centre of torsion estimated by the inverse method [40]. The spectral amplitude of ${\dot{\theta }}_{HJZ}$ is lower than that of ${\dot{\theta }}_{45}$, which is coherent with the position of the centre of rotation.

The temporary experiment was mainly conducted on ambient vibrations and in order to compare the translation and rotation amplitudes, the root mean squares (RMS) of the time windows are considered in the rest of this article.

## 4. Results and Discussion

### 4.1. Variation of Translation and Rotation Frequencies

**Figure 6:**
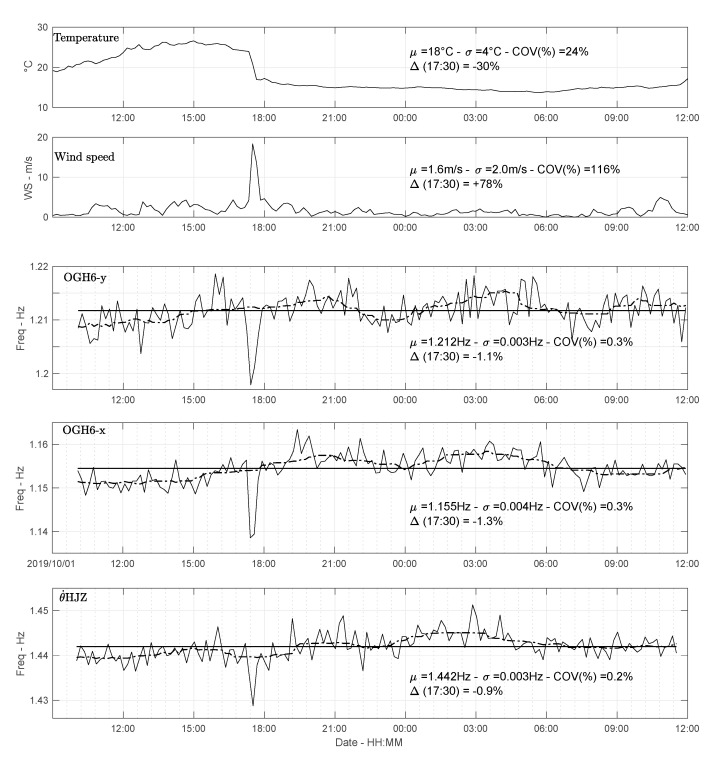
Time variation of weather conditions (temperature and wind velocity) and resonance frequencies in translation in directions x and y (station OGH6), and in rotation obtained by the rotation sensor HJZ.

During the temporary experiment, major fluctuations in temperature and wind speed were observed (Figure 6). The lateral resonance frequencies of buildings vary as a function of external loading produced by temperature and wind variation [43,44] or if the amplitude of the loading varies in amplitude [45]. Guéguen et al. [43] and Astorga et al. [45] recently confirmed that the translation frequencies in different types of buildings shift towards the low frequencies, even for the very slight deformation levels caused by earthquakes. The signature of these variations and recovery provide information relevant to the presence of defects or damage in the structure for Structural Health Monitoring (SHM).

Figure 6 shows the variation of translation and rotation resonance frequencies as a function of temperature and wind speed variations. The frequencies were obtained by applying the random decrement technique (RDT), which was originally proposed by Cole [46] to calculate damping. However, for monitoring slight frequency variations in terms of SHM and physical process interpretation, RDT can be used to enable detailed evaluation of such fluctuations [38,43]. In this study, the random decrement signature is obtained by stacking 5-s windows (approximately 5–10 periods) selected in each 10 min window. The frequency is then estimated by fitting a function of the form $e^{-\xi \omega t}$ to the signature. The frequency value thus obtained is attributed to the time in the middle of the 10 min window.

At around 17:30, a fast increase in wind speed starting at the same time as a sudden fall in temperature indicates a sudden, short storm, resulting in a significant increase in the amplitudes recorded at the top (Figure 4b). The ratio of the amplitudes during the storm and under ambient vibrations (Figure 4) is approximately 17 for rotations and 14 for translation, i.e., relatively consistent. Table 1 summarizes the values of changes in translation and rotation of modal values, as well as changes in weather conditions. Translation and rotation frequencies at 17:30 vary by approximately 1% compared with their mean values (Table 1, Figure 6), with coefficient of variation (COV) around 0.2–0.3% over 24 h. These variations are also consistent between translation and rotation, with synchronized oscillations around the average values. This indicates the possibility of obtaining a consistent value for the rotation frequencies and their variation with a sensitive rotational motion broadband sensor (BlueSeis-3A sensor), offering new perspectives in terms of rotation-based structural health monitoring.

### 4.2. Comparison of Rotation Values

**Figure 7:**
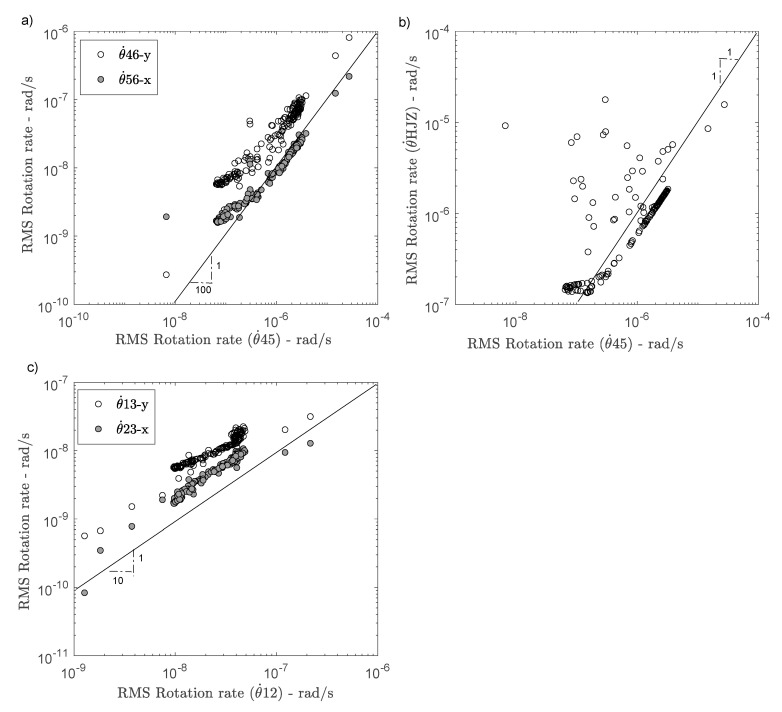
Comparison of the rotation rates calculated by the array-derived method at the top (**a**); with the rotation sensor HJZ (**b**) and at the bottom of the structure (**c**).

Figure 7 compares the RMS values of ${\dot{\theta }}_{45}$ rotations with rotations ${\dot{\theta }}_{46}$ and ${\dot{\theta }}_{56}$ (7a) and with ${\dot{\theta }}_{HJZ}$ (7b). A large amplitude difference is observed between the rotations estimated by Equations (1) and (2), depending on the pair of stations considered. By applying the finite difference method, the rotation rate for each pair of stations in directions x and y (at the top and at the bottom) gives a value at the mid-point of the line passing through the two stations. The ratios of rotations ${\dot{\theta }}_{46}$/${\dot{\theta }}_{45}$ and ${\dot{\theta }}_{56}$/${\dot{\theta }}_{45}$ are relatively constant, i.e., 0.02 and 0.01, i.e., a rotation rate at the mid-point of the line between stations OGH4-OGH6 and OGH5-OGH6 50 and 100 times less than the rotation rate between OGH4-OGH5. This must indicate the presence of shear deformation of the structure at the top. Conversely, the ratios at the bottom ${\dot{\theta }}_{13}$/${\dot{\theta }}_{12}$ and ${\dot{\theta }}_{23}$/${\dot{\theta }}_{12}$ (Figure 7c) are around 0.38 and 0.19, i.e., a rotation rate at the mid-point of the OGH1-OGH3 and OGH2-OGH3 lines 2 and 5 times lower than at the mid-point of line OGH1-OGH3. At ground level, the ratio is closer to 1/1, which confirms the infinitesimal shear deformation assumption, considering the homogeneous and isotropic wavefield (which may be a first order approximation for seismic noise). The difference between the two rotation values along lines x and y may be the consequence of a different soil-structure coupling in the two directions under weak motion, since the sensors are not in the free-field, but on the foundation. This assumption could be verified by using a rotation sensor at the bottom of the structure and one in the free-field. Incidentally, the contribution of the deformation in directions x and y at the bottom of the building (0.38/0.19 = 2) is also found at the top of the building (0.02/0.01 = 2).

With the exception of local variations of rotation ${\dot{\theta }}_{HJZ}$ (due to the temporary installation not exempt from possible noise), the comparison between rotations ${\dot{\theta }}_{45}$ and ${\dot{\theta }}_{HJZ}$ is around 1/2, due to the estimation of the rotation rate ${\dot{\theta }}_{45\;}$ at the mid-point close to centre of torsion. The BlueSeis-3A sensor thus enables a pure rotation measurement, not coupled with the translation motion. This ratio is linear and constant for the rotation values ${\dot{\theta }}_{HJZ}$ > 1.5 × 10^−7^ rad/s, which is the experimental sensitivity of the BlueSeis-3A sensor (compared with the theoretical sensitivity of 2.5 × 10^−8^ rad/s). Consolidating the experimental infrastructure could have reduced experimental sensitivity.

### 4.3. Acceleration versus Rotation and Phase Velocity

**Figure 8:**
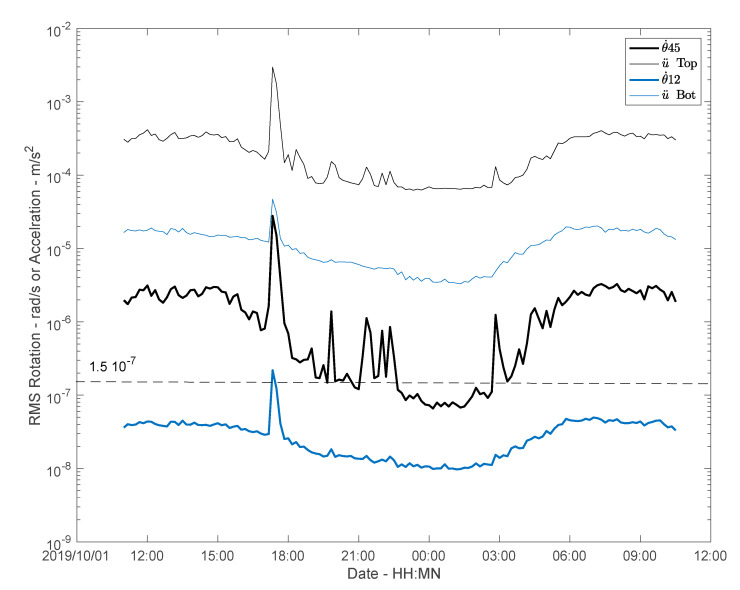
Amplitude variation of the RMS values of translation and array-derived rotations during the experiment, at the top and at the bottom of the structure. The dashed horizontal line corresponds to the sensitivity limit of the rotation sensor, estimated experimentally.

To compare translations and rotations, acceleration is calculated as the average of the acceleration RMS values recorded by the three sensors in the two directions x and y. The rotations considered are those provided by Equation (1), i.e., ${\dot{\theta }}_{13}$ and ${\dot{\theta }}_{45\;}$. The RMS value variations over time are given in Figure 8. The first observation is that rotation at the top ${\dot{\theta }}_{45}$ results in values lower than the sensitivity of the rotation sensor ${\dot{\theta }}_{HJZ}$ (1.5 × 10^−7^ rad/s) over the midnight period. At ground level, the ${\dot{\theta }}_{12}$ values fall below the detection threshold of the BlueSeis-3A, since this instrument does not enable rotation measurement at the bottom of the structure in this case (with the exception of the window covering the storm, to be compared with the accelerations produced by earthquakes in the free-field). The variations between acceleration and rotation and between top and bottom are relatively consistent. The average ratios are provided in Table 2.

**Figure 9:**
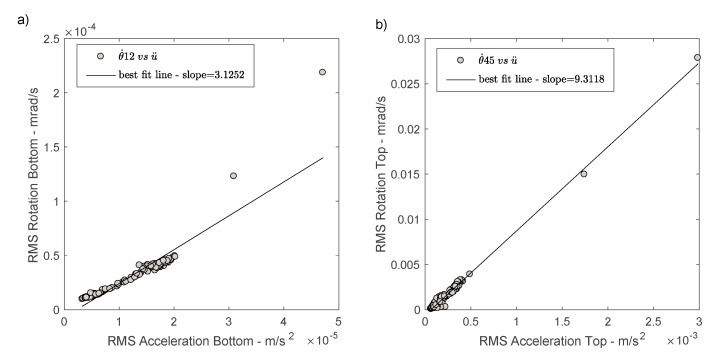
Comparison between translation and array-derived rotation at the bottom (**a**) and at the top (**b**) of the structure.

First of all, the dynamic effect of the structure response is clearly reproduced, with amplification of the translation and rotation motion between the bottom and the top. However, the variation coefficient is larger for rotation (65%) than for translation (34%), confirming the importance of the dynamic effect of rotation in the structural response. This dynamic effect is also confirmed by the ratio of accelerations and rotations at the top of the structure, with an average ratio of around 315, similar to what can be observed at the bottom (404). The amplitude ratio between rotation and translation is 10–20 times greater at the top than at the bottom. The GCH data are not sufficient to enable analysis of the relationship between the translation and rotation motion during earthquakes and thus help to evaluate the accidental torsion due to dynamic effects. However, these data can provide information on the relationship between translation and rotation under weak motion (linear elastic response) in the case of a relatively symmetrical structure, with the characteristics Ω > 1 and Fy > 1 Hz. Figure 9 shows rotation RMS values in mrad/s compared with acceleration RMS values in m/s^2^. A linear fit to the data of the form y = ax + b is applied, at the top and bottom of the structure, giving the following relationships:

(3) $${\dot{\theta }}_{12}=3.125\;\ddot{u}-0.00001$$

(4) $${\dot{\theta }}_{45}=9.312\;\ddot{u}-0.00060$$

These can be compared with the results of Takeo [47] and Liu et al. [48] for free-field earthquake data with accelerations of less than 0.3 m/s^2^ and 3 m/s^2^, respectively. The fit coefficients *a* are 1.301 [47] and 1.454 [48]. The motion at the bottom of the structure show a $\dot{\theta }$/$\ddot{u}$ ratio three times greater under ambient vibrations than under moderate earthquake conditions [47]. This ratio increases to 10 at the top, confirming the dynamic effect of rotation and the significant contribution of rotation to the overall structural response, at least under ambient vibrations. A misfit at the bottom of the structure is also observed for the two periods with the strongest accelerations (at the time of the storm), due to interaction between the soil and the structure. The data available is not sufficient to distinguish the effect of accident torsion due to rotational ground motion and due to dynamic effects. Most previous studies focused on the effects of rotational ground motion on the torsional response with favourable characteristics (e.g., Ω < 2/3 and Fy > 2 Hz in [9]; Ω < 1 and Fy > 3 Hz in [12]; Ω < 1 and Fy > 1 Hz in [49]). The overall observation is that the rotational ground motion effect is much greater in symmetrical buildings, like the GCH building. Shakib and Tohidi [49] also showed the significant effect of site conditions and soil-structure interaction.

### 4.4. Phase Velocity Derived from the Rotation Measurement

Seismic interferometry is a powerful technique based on the combination of signals recorded by different sensors to estimate the response of the medium (e.g., [50,51,52,53,54]). This technique is based on the correlation of waves recorded by different receivers throughout the height of a building in our particular case. When wave excitation is uniformly distributed in the space or between the normal modes of the system, it is possible to demonstrate that this correlation corresponds to the Green’s function, which explains wave propagation between the receivers [51]. Using the sensor at the top of the building as a reference, the result is an up-going and down-going impulse wave through the vertical array of the building, characterizing the dynamic response of a fixed base type structure. Since the seminal paper published by [51], several authors have applied seismic interferometry by deconvolution (SIbyD) to actual buildings to understand their elastic response with or without soil-structure interaction, or to monitor their structural health during strong earthquakes by evaluating variations in wave velocity (e.g., [25,55,56,57,58]).

Using experimental data, the continuous beam theory can be considered to interpret the wave propagation characteristics for the building response assessment ([25,55,56,57,58]). Based on this beam model, Rahmani and Todorovska [25] and Michel and Guéguen [58] interpreted the nature of the waves obtained by SIbyD (evanescent, dispersive or permanent regime) and the experimental values of the impulse wave velocity for specific buildings according to their dynamic response. Guéguen et al. [57] recently demonstrated the relationship between impulse wave propagation velocity and the structural response for different beam models representing structures (i.e., dispersive and non-dispersive beam models) by calculating the dispersion curves and the variation of phase velocity as a function of structure model (in relation with the shear-to-bending ratio).

**Figure 10:**
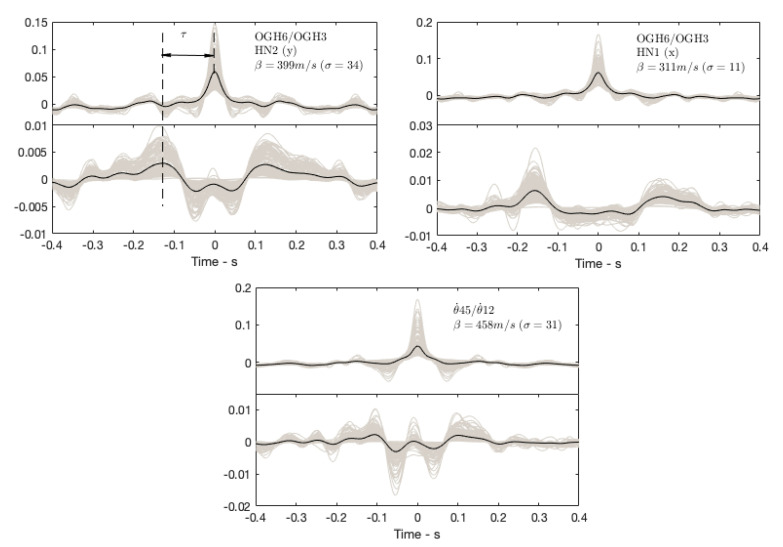
Interferograms obtained by seismic interferometry by deconvolution (SIbyD) between the horizontal components of sensors OGH6 and OGH3 in directions x and y, and with the array-derived rotation rates at the top ${\dot{\theta }}_{45}$ and at the bottom ${\dot{\theta }}_{12}$ of the structure.

Figure 10 shows the average of the interferograms calculated on 10 min windows, band-pass filter 0.5–5 Hz, to take into account the compromise between frequency resolution and time resolution [56] considering horizontal components at stations OGH6 (top) and OGH3 (bottom) in both directions x and y, and the rotation components at the top and bottom estimated by Equation (1) (i.e., ${\dot{\theta }}_{45}$ and ${\dot{\theta }}_{12}$). The velocity of impulse wave β is calculated by the ratio between building height (H = 53 m) and the time delay of the impulse between the bottom and the top (β=H/τ).

Velocity is greater (β = 399 m/s) in the y direction than in the x direction (β = 311 m/s) due to building design, consistent with the different resonance frequencies in the two directions (Figure 5). Velocity is higher (β  = 458 m/s) in rotation. Note that the velocity variation is significant, with a coefficient of variation between 5 and 10% regardless of the direction considered.

In free-field, the phase velocity is derived from the rotational and translational ground motion recordings (e.g., [20,59], as follows:

(5) $$C=\frac{1}{2}\frac{\ddot{u}}{\dot{\theta }}$$

where *C* is the phase velocity.

**Figure 11:**
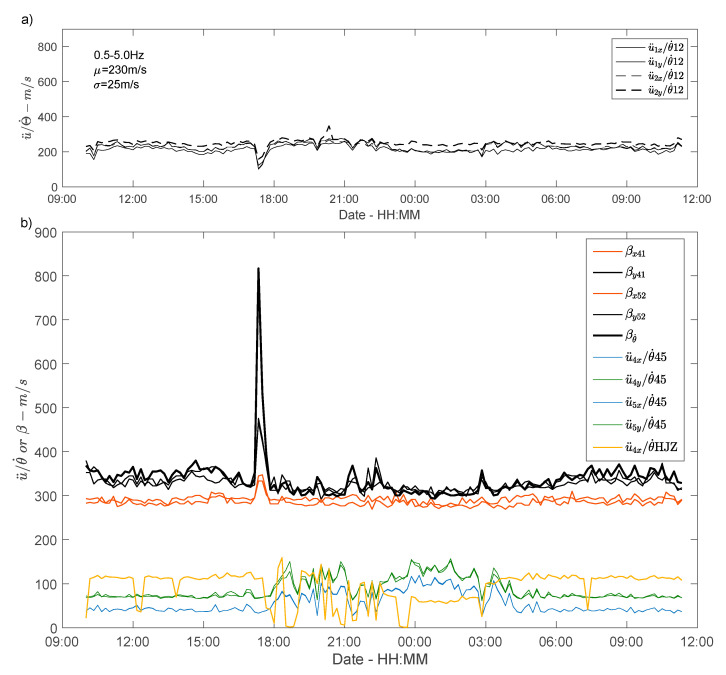
(**a**) Phase velocity variations calculated from the ratio between acceleration and array-derived rotation rate at the bottom of the structure; (**b**) Comparison of velocities calculated by SIbyD and derived from the ratios between acceleration and rotation at the top of the building.

Figure 11a shows the velocity variation as a function of time, calculated at the bottom of the building using Equation (5), considering the different RMS values of the rotation rate ${\dot{\theta }}_{12}$ and acceleration in directions x and y of sensors OGH1 (${\ddot{u}}_{1x}$ and ${\ddot{u}}_{1y}$) and OGH2 (${\ddot{u}}_{2x}$ and ${\ddot{u}}_{2y}$) (bandpass filter 0.5–5 Hz). The velocity values oscillate around 230 m/s (COV = 11%). This value is to be compared with the S wave velocity (Vs) of the uppermost soil layer (approximately 200 m/s) associated with a site resonance frequency of 2 Hz in the vicinity of Grenoble city hall. We observe between 0.5 and 5 Hz, i.e., within the frequency band corresponding to the structural resonance frequencies in translation and in torsion, the effect of the soil-structure interaction with a velocity decrease to 150 m/s corresponding to the time of the storm (17:30).

At the top of the building (Figure 11b), the effect of the storm is also clearly visible on the velocities calculated by SIbyD. Excluding the windows around 17:30, the velocities calculated by SIbyD give average values of $\beta_{x41}$ and $\beta_{x52}$ 292 m/s, $\beta_{y41}$ and $\beta_{y52}$ 322 m/s and $\beta_{\dot{\theta }}$ 352 m/s in directions x, y and z, respectively, with a slight fluctuation between day and night for directions y and z. These velocities are stable regardless of the pair of stations considered (OGH4/OGH1 and OGH5/OGH2 in this case). The velocities increase significantly when the storm windows are considered (Figure 11b), reaching peak values at 17:30 of 345 m/s, 475 m/s and 816 m/s in directions x, y and z, respectively. Considering the conventional relationship between resonance frequency and velocity, it is difficult to interpret this velocity increase corresponding to a frequency reduction (Figure 6) when loading increases. However, according to [50], the SIbyD approach considers input loading at the bottom of the structure, which is not the case here during the storm. This interpretation will be verified in further studies, using the earthquakes data recorded by the permanent array of the city hall building.

Comparing the translation accelerations and rotation rates (Equation (4)) derived from the array at the top or provided by the rotation sensor, a significant difference is observed between the values. Some general characteristics are certainly coherent, like the same values and the same variations observed over time whatever the considered stations for acceleration (OGH4 and OGH5), or the higher velocity (around 67 m/s) calculated with the y accelerations compared with the x accelerations (42 m/s). With the rotation sensor, even if the values show large transient fluctuations, probably due to the temporary, non-consolidated installation of the rotation sensor, the velocities are around 105 m/s. For rotation values derived from the translation array and those from the rotation sensor (here, only ${\ddot{u}}_{4x}/{\ddot{\theta }}_{HJZ}$ is considered, since the other components give the same results), we also observe that during the night immediately after the storm, the velocity values are more dispersed, although no satisfactory interpretation can be proposed.

The difference between the velocities by interferometry and using the acceleration versus rotation components ratio can be explained by the fact that they do not measure the same velocities. For example, for a structure exhibiting bending behaviour (type Euler-Bernouilli), Guéguen et al. [57] demonstrated a large difference between the phase velocities and the impulse wave velocities obtained by SIbyD, depending on the frequency bands considered. Ebrahimian et al. [56] also showed that the velocity values obtained by SIbyD are between the phase and group velocity values. Again, earthquake data recorded by the permanent array of the city hall building might provide insight on the building velocity values derived from rotations and accelerations.

## 5. Conclusions

We have shown that a rotation sensor can be used to provide relevant information on the elastic response of the structure. Comparison of the rotation rates derived from the array or provided by the rotation sensor shows that the calculation of rotation in structures using station pairs aligned in the main directions underestimates the rotation rates. When the centre of torsion is located on this same line, the rotation rate is similar to that obtained by the rotation sensor considered in this study. Measuring rotation at different location on the top floor could improve the observation of the translation/rotation coupling and the assessment of the torsion centre. Finally, with an array of rotation sensors installed at several floors, operational modal analysis tools might provide information about the torsional mode shape of the building.

Under ambient vibrations, a large ratio is observed at the ground level between acceleration and rotation compared with even moderate earthquake conditions (e.g., [47]). This observation confirms the dynamic effect on accidental eccentricity observed by various authors and offers information on the linear–non-linear transition. It would be useful to share a large amount of data recorded in buildings from a large number of earthquakes with different magnitude using an array comprising station pairs or rotation sensors to confirm and provide more explanations in this issue.

Although many questions remain unanswered, the synchronized measurement of rotation and acceleration provides interesting elements for the estimation of the dynamics of structure, in terms of both response and properties. Effort remains to be done, but the estimation of phase velocities with rotation, interpreted by a continuous beam type building model, suggests potential applications of interest in the fields of earthquake response of structures, identification of property variations by phase velocity inversion and, more generally speaking, seismic structural health monitoring. For example, passive seismic monitoring of structures assesses the structural health, by combining detection and spatial localization of damage. The latter is based for example on the spatial derivatives of modal forms [60] or on the perturbation-based inverse problem [61], which could thus become more efficient by complementing by the rotation modes the classical assessment done with the translation modes. Before that, additional studies must be defined, on the required sensitivity of the rotation sensors for the monitoring of buildings or the theoretical interpretation of the measured rotations with respect to the structural health. This could be achieved by the multiplication of rotation sensors in civil engineering structures.

The recording, analysis and interpretation of torsion (and more generally rotations along three axes) observed in structures and its contribution to the overall response under earthquake conditions are also essential to the future development of earthquake engineering design codes. This study concerns ambient vibrations, but with earthquakes, if the contribution of rotation to structural drift is not correctly estimated, we might incorrectly assume that the drift observed results entirely from the translation displacement of the structure, which might lead to undue confidence in the conservatism of current design methods. The generalization of instrument deployment in structures, designed to focus on rotation, and the use of rotation sensors can be expected to provide essential information on the seismic response of structures, far beyond the data that can be obtained from even the most sophisticated numerical models. The community should support any effort in this field.

## Figures and Tables

**Table 1:** Characteristics of the variations of the weather parameters and modal parameters (Δ corresponds to the variations during the local storm).

Parameters		μ	σ	σ/μ	Δ
Temperature °C		18	4	24%	−30%
Wind speed m/s		1.6	2	116	78%
Frequency Hz	y-dir	1.212	0.003	0.3%	−1.1%
	x-dir	1.155	0.004	0.3%	−1.3%
	z-dir (${\dot{\theta }}_{HJZ}$)	1.442	0.003	0.2%	−0.9%

**Table 2:** Average ratios of root mean square (RMS) values between translation acceleration and torsion rate at the top and at the bottom of the structure.

RMS Ratio	Mean	Std	COV (%)
$\ddot{u}$ Top/Bot	18.3	6.3	34
${\dot{\theta }}_{45}$/${\dot{\theta }}_{12}$	40.6	26.4	65
u Top/${\dot{\theta }}_{45}$	315.3	261.1	83
u Bot/${\dot{\theta }}_{12}$	404.5	38.4	9

## Data Availability

The accelerometric and weather data presented in this study are openly available in RESIF-DC (http://seismology.resif.fr/) at http://dx.doi.org/10.15778/RESIF.RA. The Rotation Data are Available on Request to Philippe Gueguen.
